# Supplementary material for: Addition of Lactobacillus fermentum to Fermented Sea Buckthorn (Hippophae rhamnoides L.) Fruit Vinegar Significantly Improves Its Sour Taste
Source: Foods. 2025 Mar 31;14(7):1223. doi: 10.3390/foods14071223 (PMC11988584; doi:10.3390/foods14071223)
Supplement: Supplementary file 1 [file foods-14-01223-s001.zip › Supplementary Figure S1-S3.pdf]

Supplementary Materials

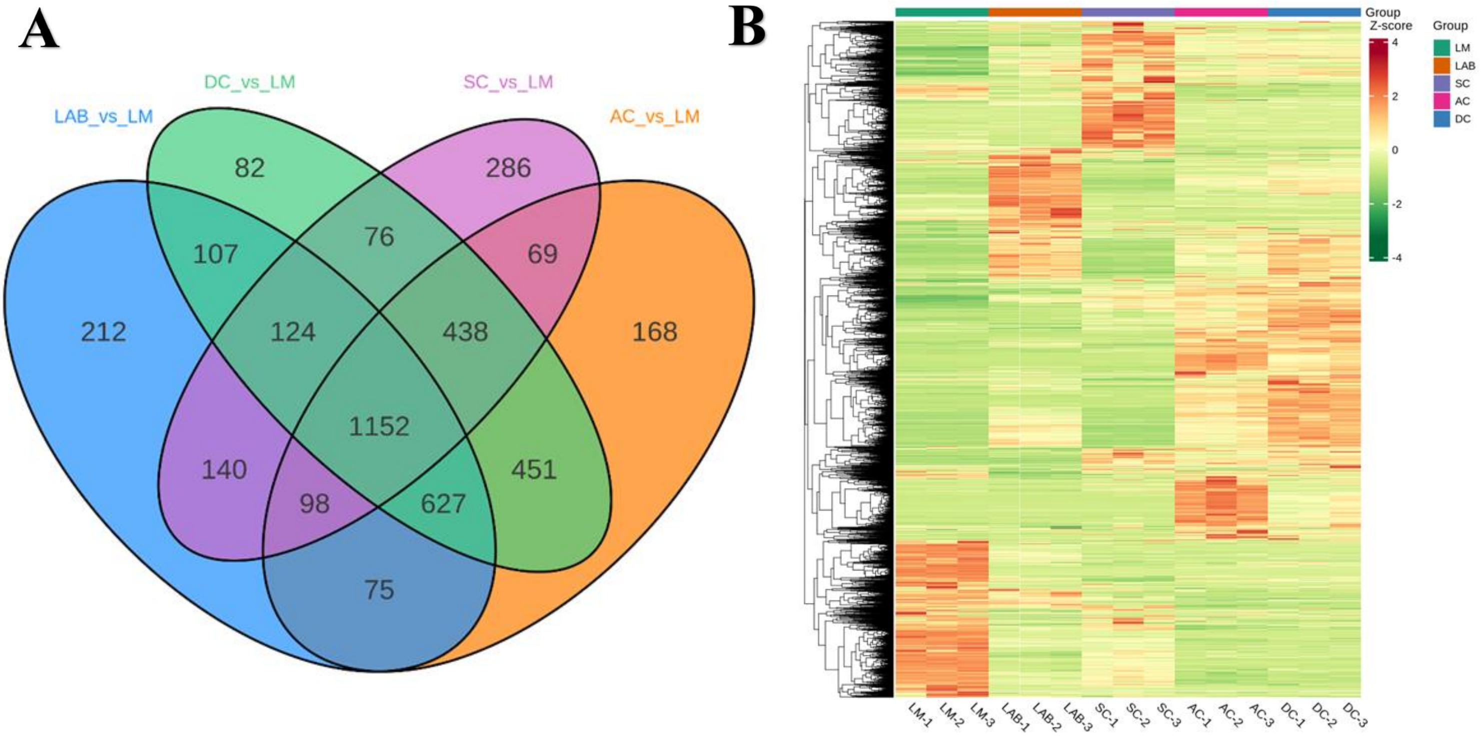

Figure S1. Hierarchical cluster analysis plots of sea buckthorn fruit vinegar at different fermentation stages. **A**: Metabolite Wayne diagram. **B**: Metabolite clustering heat map. (Horizontal coordinates show sample names, vertical coordinates show differential metabolites. The color shift from green to red represents the relative abundance of metabolite expression from low to high).

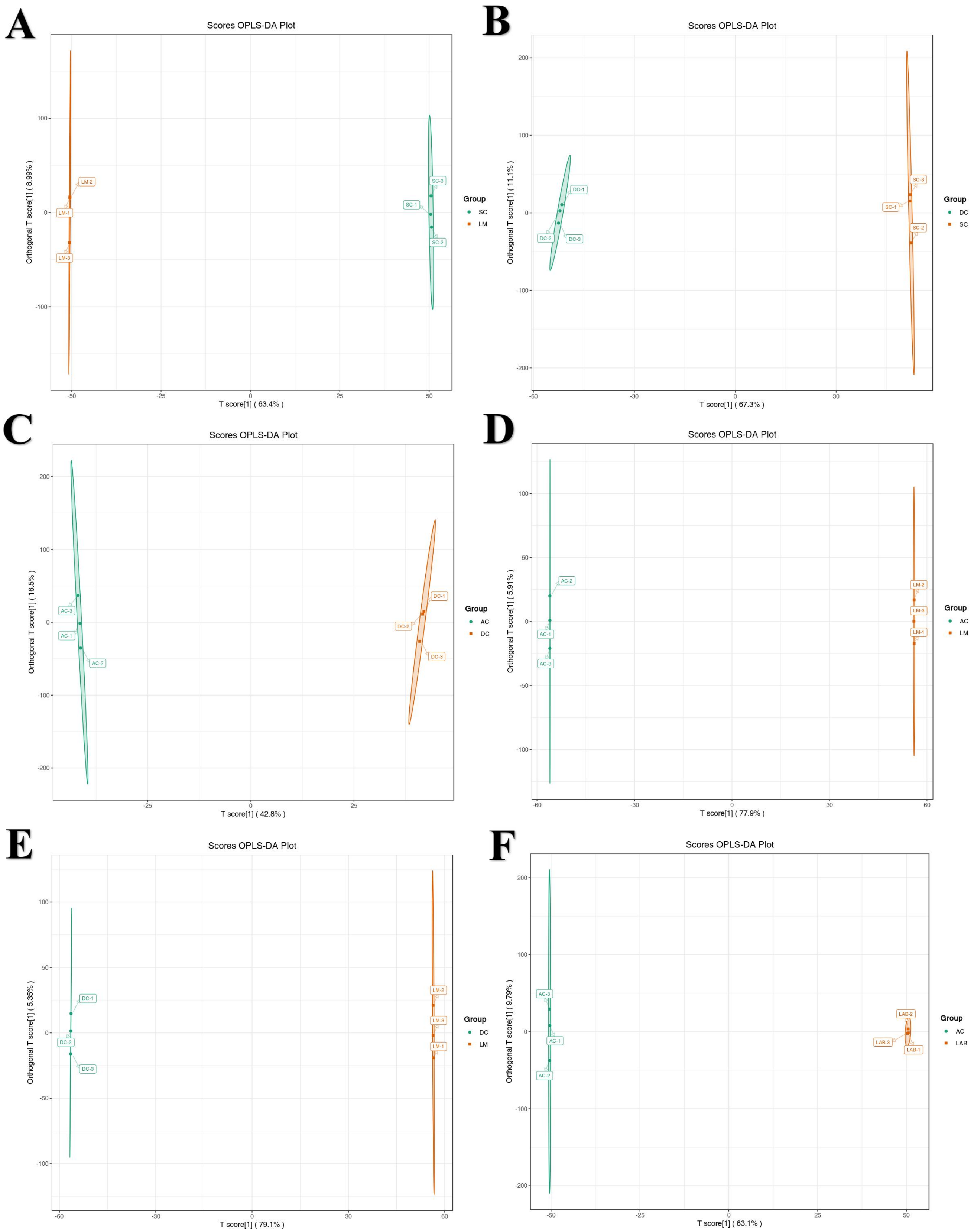

Figure S2. Plot of OPLS-DA analysis comparing different fermentation stages. **A**: SC vs LM; **B**: DC vs SC; **C**: AC vs DC; **D**: AC vs LM; **E**: DC vs LM; **F**: AC vs LAB.

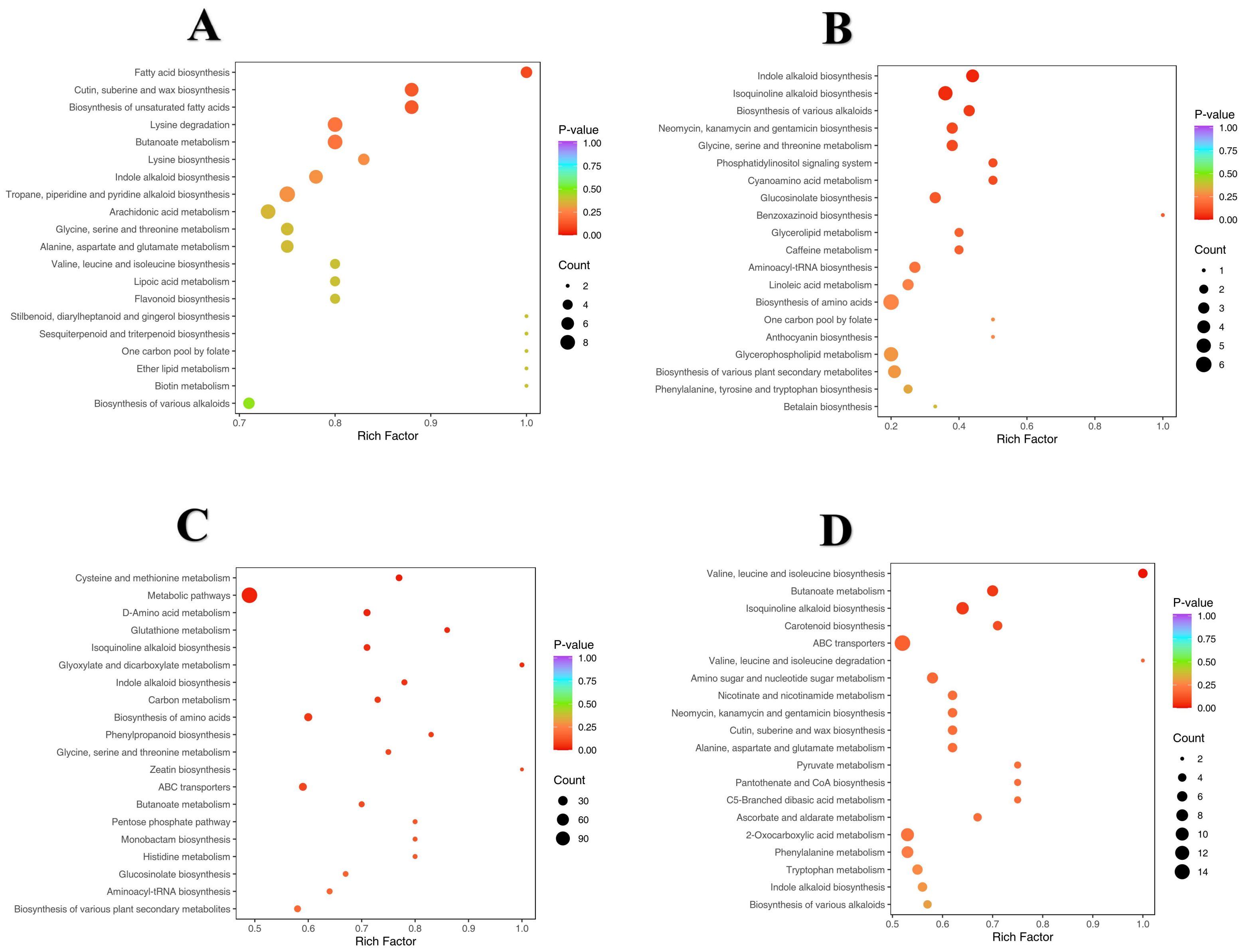

Figure S3. Enrichment analysis of differential metabolite pathways. **A**: AC vs LM; **B**: AC vs DC; **C**: AC vs SC; **D**: AC vs LAB. Colors indicate p-values and bubbles indicate the amount of enrichment of differential metabolites.
