# Supplementary material for: Addition of Lactobacillus fermentum to Fermented Sea Buckthorn (Hippophae rhamnoides L.) Fruit Vinegar Significantly Improves Its Sour Taste
Source: Foods. 2025 Mar 31;14(7):1223. doi: 10.3390/foods14071223 (PMC11988584; doi:10.3390/foods14071223)
Supplement: Supplementary file 1 [file foods-14-01223-s001.zip › Supplementary information Note S1.pdf]

Supplementary information Note S1

Information on the strains used in the fermentation process

Lactobacillus fermentum F:Characteristics: The bacterium is short rod-shaped, Gram-positive, does not produce spores. It has strong ability to metabolize carbohydrates to produce acid, can synthesize glucan and heteropolysaccharide, and can ferment sugar to produce lactic acid or other acids. The total acid content of fermented fruit juice can reach 3.5mg/kg. Genbank Serial No.:MZ674413.Strain reference use: acid production, fermented fruits and vegetables research. Strain origin: CHINA CENTER OF INDUSTRIAL CULTURE COLLECTION. strain number: CICC 25124. latin name: *Lactobacillus fermentum*. Isolated Substrate: Zhedong traditional pickled winter melon. Pathogenicity object: none.

Saccharomyces cerevisiae RV: Characteristics: Fermentation of glucose, sucrose, maltose, galactose, and raffinose; non-fermentation of lactose, saccharose, and soluble starch; non-fermentation of xylose, ethanol, and glycerol; production of ethyl acetate. Strain origin: CHINA CENTER OF INDUSTRIAL CULTURE COLLECTION. strain conservation number: CICC 1263. latin name: *Saccharomyces cerevisiae*. reference use: making fruit wine. Pathogenicity object: none.

Acetobacter pasteurianus PAC: Characteristics: colonies beige, irregular, 1.0-1.5 mm, produce hyaline rings on agar with calcium carbonate. Cells rod-shaped, slightly curved, single; G-, do not form bud cells, organic chemical energy heterotrophic, PH5.4-6.3; not from D-arabinose, fructose, lactose, maltose rhamnose, sucrose, cotton candy or starch acid production; easy to produce degenerate, some strains have pods or produce mucus material. Acetic acid production. Reference use: brewing vinegar, apple cider vinegar production. Strain origin:CHINA CENTER OF INDUSTRIAL CULTURE COLLECTION. Strain conservation number: CICC 20001. Latin name: *Acetobacter pasteurianus*. Pathogenic object:None.
