# Supplementary material for: Addition of Lactobacillus fermentum to Fermented Sea Buckthorn (Hippophae rhamnoides L.) Fruit Vinegar Significantly Improves Its Sour Taste
Source: Foods. 2025 Mar 31;14(7):1223. doi: 10.3390/foods14071223 (PMC11988584; doi:10.3390/foods14071223)

Supplementary information Note S2

Total flavonoids standard curve

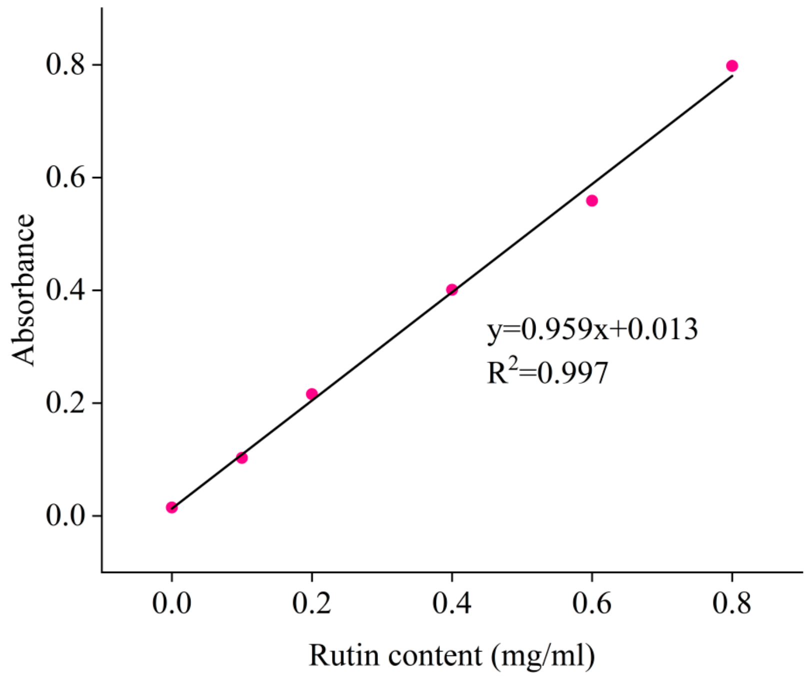

Total Phenol Standard Curve

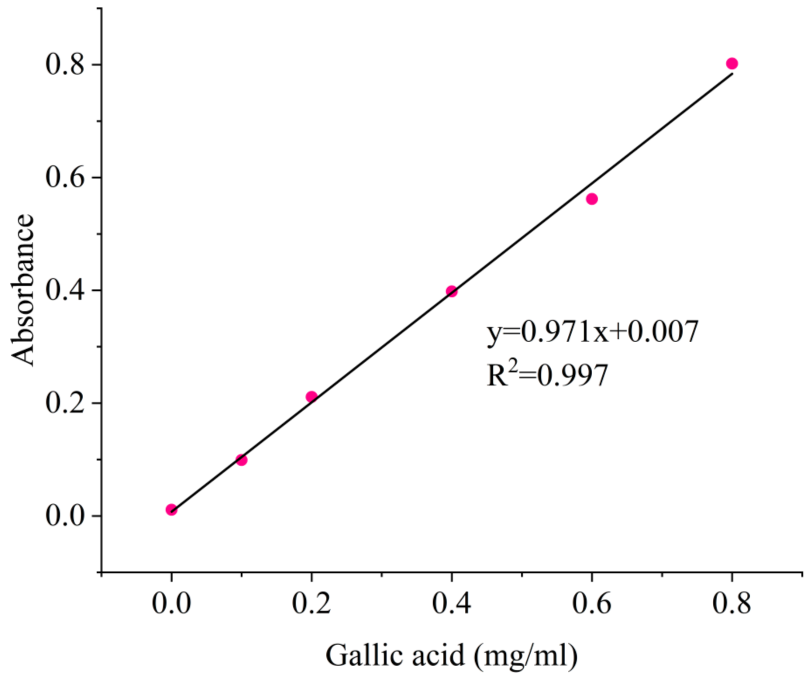

Supplement: Supplementary file 1 [file foods-14-01223-s001.zip › Supplementary information Note S2.pdf]
